# Comparative Efficacy of Vibration foam Rolling and Cold Water Immersion in Amateur Basketball Players after a Simulated Load of Basketball Game

**DOI:** 10.3390/healthcare11152178

**Published:** 2023-07-31

**Authors:** Fengping Li, Yang Song, Xuanzhen Cen, Dong Sun, Zhenghui Lu, István Bíró, Yaodong Gu

**Affiliations:** 1 Faculty of Sports Science, Ningbo University, Ningbo 315211, China; 2 Doctoral School on Safety and Security Sciences, Óbuda University, 1034 Budapest, Hungary; 3 Faculty of Engineering, University of Szeged, 6720 Szeged, Hungary

**Keywords:** amateur basketball player, post-game recovery, cold water immersion, vibration foam rolling, sports biomechanics

## Abstract

To compare the efficacy of different recovery strategies (sitting; cold water immersion, CWI; vibration foam rolling, VFR) on the lower extremities of amateur basketball players after the simulated load of a basketball game, we assessed the power, agility, and dynamic balance before and after interventions. Ten amateur basketball players alternately underwent 12 min of sitting, 12 min of CWI at 5 °C, and 12 min of VFR. The power, agility, and dynamic balance were measured immediately post-warm-up, immediately post-game, immediately post-intervention, 1 h after interventions, and 24 h after interventions. To simulate the load of a basketball game, specific movements were designed and implemented. Jump height was measured using a Kistler force plate. Reaction time and dynamic balance score were assessed using the Pavigym agility response system and the Y balance test, respectively. The data were analyzed with a two-way repeated measures analysis of variance (ANOVA). The results showed that the vertical jump height significantly decreased after the CWI intervention compared to the CON and VFR groups (*p* < 0.001). At 1 h after the intervention, the vertical jump height in the CON group showed delayed recovery compared to the CWI and VFR groups (*p* = 0.007; *p* < 0.001). At 24 h after the intervention, the vertical jump height in the CWI group further increased and was significantly different from the CON and VFR groups (*p* < 0.001; *p* = 0.005). Additionally, reaction times significantly increased immediately after the CWI intervention (*p* = 0.004) but showed further recovery at 24 h compared to the CON group (*p* < 0.001). The dynamic balance score significantly rebounded after the CWI intervention compared to the CON group (*p* = 0.021), with further improvement at 24 h (*p* < 0.001). CWI initially showed negative effects, but over time, its recovery effect was superior and more long-lasting. VFR had the best immediate effect on lower limb recovery after the game.

## 1. Introduction

In both elite and recreational basketball sports, athletes frequently participate in games on consecutive days, often multiple times per week. These games involve high physical demands, including various moderate- to high-intensity activities such as explosive jumps and rapid accelerations, decelerations, and changes in direction during the offensive and defensive phases [1,2]. It is well-documented that an imbalance between training/competition load and recovery can lead to the excessive accumulation of fatigue over time, potentially impairing subsequent training and performance [3,4,5]. Therefore, a wide range of post-exercise recovery strategies are implemented to mitigate the detrimental effects of fatigue and maintain optimal performance. These strategies include static or dynamic stretching, active recovery, massage, the use of compression garments, and nutritional supplementation [6,7].

Cold water immersion (CWI) is currently a widely used recovery modality and has become an essential component of post-exercise routines in many team sports [8]. It involves immersing some or all of the athlete’s body in cold water baths for several minutes [9]. CWI is believed to reduce skin, muscle, and core temperature, increase hydrostatic pressure, improve venous return through vasoconstriction, and promote enhanced recovery [10,11]. Several studies have demonstrated the benefits of CWI on various aspects of recovery in basketball. Similarly, the research focused on elite soccer players has shown significant enhancements in sprinting performance [12]. While CWI protocols using immersion times of 10–15 min at temperatures ranging from 11 °C to 15 °C are considered the standard for immediate and delayed recovery effects [13], the influence of water temperature on CWI remains inconclusive. Daniel et al. [14] compared the effects of CWI using 14 °C (CWI_14°C_) or 5 °C (CWI_5°C_) on team sport players’ recovery and found that CWI_5°C_ was more effective in restoring peak power output. This suggests that water temperature may have an impact on the effectiveness of CWI for recovery. However, since few studies have investigated the recovery effects of CWI with water temperatures below 10 °C (as 75% of studies typically range from 11 °C to 15 °C) [15], and these positive findings have mainly focused on elite players rather than non-elite, further research is needed to explore the recovery effects of CWI (with water temperature < 10 °C) on amateur players.

While CWI remains a commonly used recovery technique, other modalities, such as foam rolling and vibration foam rolling (VFR), are gaining popularity as alternative methods for post-training and post-game recovery [16,17,18,19]. Foam rolling involves using body weight to apply direct pressure to targeted muscles using a foam roller, while VFR combines foam rolling with localized vibration [16,20]. Both foam rolling and VFR were shown to improve recovery, but the combination of these methods appears to offer greater benefits in the recovery process. In a study by Romero et al. (2019) [4], the effects of foam rolling and VFR on recovery following exercise-induced muscle damage were compared. The findings indicated that VFR was more effective in reducing pain perception and improving passive hip extension range in the short term. Similarly, Marina et al. (2019) [21] found a greater, though not statistically significant, recovery effect in ankle dorsiflexion, the anterior reach of dynamic balance, and perceived ankle stability after VFR compared to foam rolling. Recent studies suggest that using VFR for 90 s on a muscle group at a frequency of 30 Hz can provide benefits [16,20,22,23]. However, further research is needed to determine the optimal dosage of VFR and its specific effects, particularly in the context of recovery.

Despite the increasing popularity and widespread use of CWI and VFR, there is still a lack of research directly comparing the effectiveness of these two recovery methods, specifically in the context of amateur young basketball players. Furthermore, many studies in this field utilize heavy eccentric exercise to induce fatigue, which may not accurately reflect the recovery needs of athletes in sport-specific settings [24,25]. Therefore, this study aimed to compare the efficacy of CWI and VFR as post-game recovery strategies in amateur basketball players following simulated basketball games.

## 2. Materials and Methods

### 2.1. Participant

Ten male amateur basketball players from Ningbo University were recruited as participants for this study, the information of the participants is shown in Table 1. The eligibility criteria were as follows: first, being a starting basketball player without any professional training experience, and second, only competing in school- or university-level games 1–2 times per week and self-training 1–2 times per week; In addition, all participants were free from lower limb injuries and surgery at least 6 months before this test. Written consent was obtained after all of them were informed of the requirements of this trial. This study was approved by the Ethics Committee of Ningbo University following the Helsinki Declaration.

### 2.2. Experimental Setup

#### 2.2.1. Basketball Game Simulation

**Figure 1:**
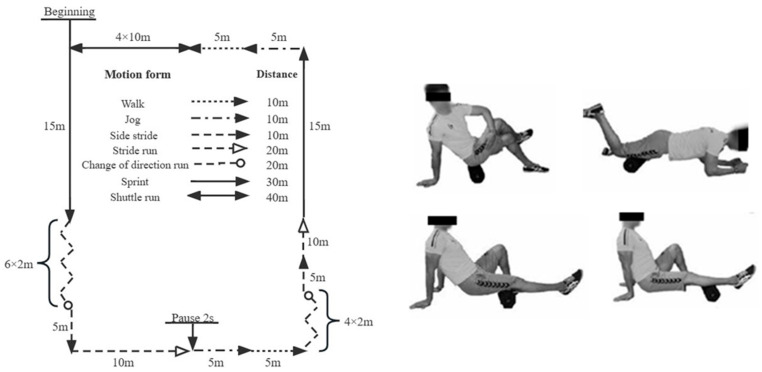
The simulated basketball games and protocols for VFR.

A formal basketball game consists of 4 quarters, with each quarter lasting 10 min. The rest time between the first and second quarters, as well as between the third and fourth quarters, is 120 s, and the halftime break is 10 min. The core players in a single game typically cover a distance of 5400–6000 m [26]. Moreover, the load of a basketball match is primarily assessed based on the players’ movement speed. The previous research has shown that a core basketball player engages in high-intensity movements for approximately 35% of the game, moderate-intensity movements for around 55%, and low-intensity movements for approximately 10% [27,28]. Thus, the time of the simulated basketball game in this study was strictly based on the official game time, incorporating the basic lower body movements of the basketball game, where one cycle consisted of a 10 m walk, 10 m jog, 10 m side stride, 20 m stride run, 20 m change of direction run, 30 m sprint, and 40 m shuttle run (Figure 1) [29,30]. So, the participants were required to complete one cycle within one minute. Each set comprised 10 cycles, and the participants completed a total of 40 sets (4 × 10) of simulated basketball game movements, covering a distance of 5600 m. The rest intervals were consistent with those in formal matches.

#### 2.2.2. Experimental Intervention Methods

A within-group crossover design was used in this study to investigate the effects of three different intervention methods on lower limb recovery after basketball games in amateur basketball players. We treated sitting as the control group (CON); after a simulated basketball game, subjects were randomly selected from three intervention modalities (CON, CWI, VFR) for the recovery intervention, and all subjects received the CON, CWI, and VFR interventions alternately during the subsequent experimental sessions. To minimize inter-group interference and the effect of training, the room temperature was controlled between 22–25 °C, all experimental tasks were scheduled to take place at the same time (9 a.m.–11 a.m. or 2 p.m.–4 p.m.), and the interval between each intervention experiment was 7 days or more, but all intervention experiments needed to be completed within a month [31]. Otherwise, to eliminate the influence of pre-experimental preparation tests on the formal experiment, the formal experiment was conducted 24 h after the pre-experimental preparation. The warm-up consisted mainly of 5 min of power cycling (70 rpm, 80 W), three repetitions of the basketball game movements in the simulation (speed increasing), and three 20 m sprints. In addition, a random draw was made to determine how subjects would receive each intervention, as described below.

Sitting: subjects were seated for 12 min without any recovery intervention after completion of the simulated basketball game.

Cold water immersion (CWI): pre-cooling acclimatization was performed first for 10–20 s, then entering the cold therapy pool (180 × 140 × 72 cm) with sports tights for cold water immersion below the waist, remaining in a sedentary state for 12 min at a controlled temperature of 5 ± 1 °C [32]. The water temperature was monitored in real-time with an electronic thermometer (TP-199, Parkoo, Ningbo, China), and ice was put into the cold therapy pool every 2 min to control the water temperature.

Vibration foam rolling (VFR): this study used the Pi Roller VFR (30 × 15 × 15 cm^3^; Vyper, Hyperice, Irvine, CA, USA), and 30 Hz was selected as the vibration frequency. The participants placed the VFR under the target muscle group (gluteus maximus, quadriceps, biceps, and gastrocnemius), rolling each muscle for 30 s/group × 3 groups for 12 min [33,34]. The vibration rolling methods for each muscle are shown in Figure 1.

### 2.3. Data Collection

The data were collected at five time points: immediately after warm-up, immediately after games, immediately after interventions, 1 h after interventions, and 24 h after interventions to test the abilities of power, agility, and dynamic balance [31,35,36].

**Figure 2:**
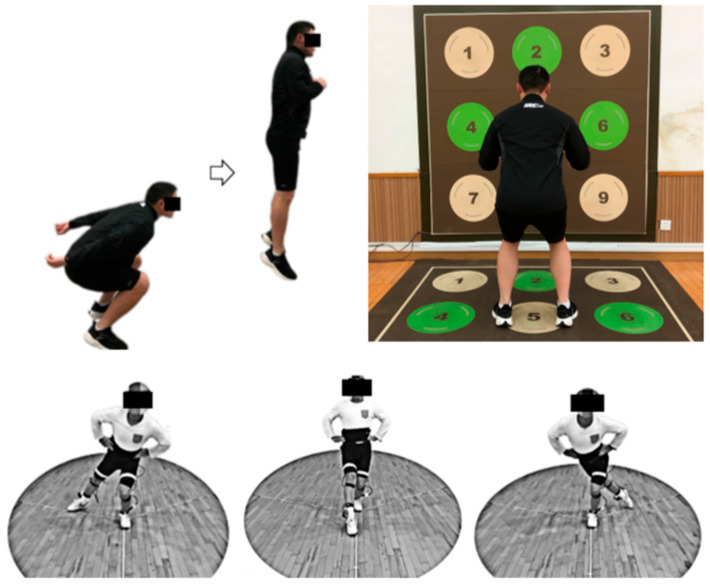
Methods of measuring physical fitness include the vertical jump Pavigym agility response system and the Y balance test.

Explosive power: this study evaluated the change in lower limb explosive strength by using the height of the reverse vertical jump. The subject stood on a Kistler force plate with the dominant leg, looking forward and swinging the hands naturally to complete the jump and landing (Figure 2). The test was carried out 3 times, and the average of the test results was taken to calculate the height of the vertical jump.

Agility: in this study, the changes in the agility of subjects before and after recovery were evaluated by reaction time. The agility test was carried out with the Free Training module of the Pavigym agility response system. The test consisted of three trials, and the average value was calculated to determine the reaction time.

Dynamic balance ability: the score of the Y balance test (Figure 2) was used to evaluate the change in the subjects’ dynamic balance ability. The main three directions of testing are anterior, posterolateral, and posteromedial (all at a 120° angle) [37,38]. The final score was calculated by the composite score formula of the Y balance test to evaluate the subject’s dynamic balance ability (Equation (1)) [39].

(1) $$\mathrm{Composite}\;\mathrm{Score}=\frac{L_{\mathrm{anterior}}+{\;L}_{\mathrm{posteromedial}}+{\;L}_{\mathrm{posterolateral}}}{3\times {\;L}_{\mathrm{posterolateral}}}\;\times \;100\%$$

### 2.4. Statistical Analysis

The data collected in this study were statistically analyzed by SPSS 21.0 statistical software (SPSS Inc., Chicago, IL, USA) using 3 × 5 two-way repeated measures ANOVA and were expressed as mean ± standard deviation (Mean ± SD). First, a box plot was used to determine whether each group of data was normal, and a Shapiro–Wilk test was used to determine whether each group of data obeyed an approximately normal distribution. A two-way repeated measures ANOVA with 3 (intervention modality: CON, CWI, VFR) × 5 (time: post-warm-up, immediate post-game, immediate post-intervention, 1 h post-intervention, 24 h post-intervention) was used to assess changes in explosive power, agility, and dynamic balance of subjects at different intervention modalities and different time points. If the data did not satisfy Mauchly’s test of sphericity, the Greenhouse–Geisser method was used to correct this and to determine again whether the effect of the interaction term on the dependent variable was statistically significant. When the effect was statistically significant, the individual effects of the factors within the study were analyzed individually and followed up with Bonferroni two-by-two comparisons by post hoc analysis. If there was no statistical significance, the main effects of the factors within the study subjects needed to be analyzed. When a main effect existed, then a two-by-two comparison was made. The significance level for this study was set at *p* < 0.05.

## 3. Results

### 3.1. Effect of Different Interventions on Explosive Power

**Figure 3:**
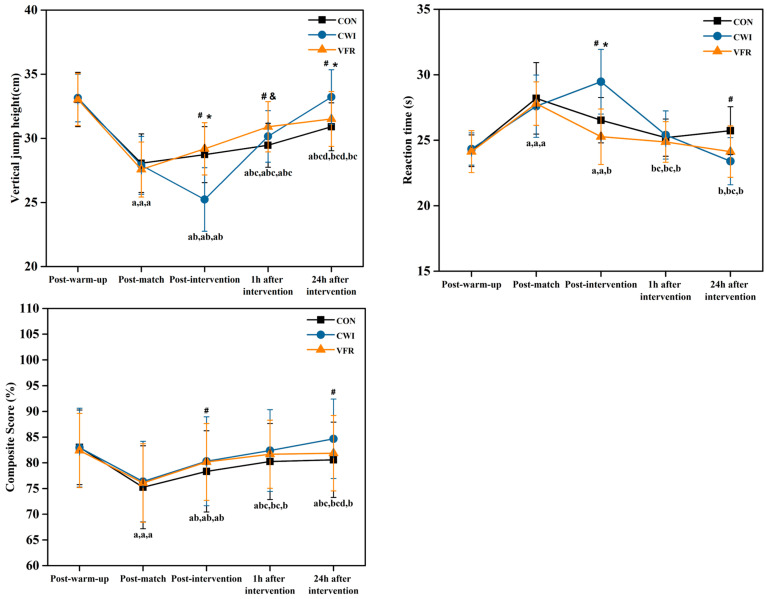
Methods of measuring physical fitness include changes in power in different interventions at different times; changes in agility in different interventions at different times; changes in dynamic balance in different interventions at different times. CON = control group; CWI = cold water immersion group; VFR = vibration foam rolling group; a. significant difference within-group for the post-warm-up in each group compared to other moments (immediate post-game, immediate post-intervention, 1 h post-intervention, 24 h post-intervention); b. significant difference within-group for immediate post-game in each group compared to other moments (immediate post-intervention, 1 h post-intervention, 24 h post-intervention); c. significant difference within-group for immediate post-intervention in each group compared to other moments (1 h post-intervention, 24 h post-intervention); d. significant difference within-group for 1 h post-intervention in each group compared to 24 h post-intervention; #—significant difference between CON and CWI; &—significant difference between CON and VFR; *—significant difference between VFR and CWI.

As shown in Figure 3 and Table 2, the height of the vertical jump after the CWI decreased significantly and was significantly different from both the CON as well as the VFR (*p* < 0.001; *p* < 0.001). At 1 h after the intervention, the delayed recovery of the height of the vertical jump after CON was significantly different from both CWI as well as VFR (*p* = 0.007; *p* < 0.001). At 24 h after the intervention, the height of the vertical jump after CWI rose further and was significantly different from both the CON and VFR groups (*p* < 0.001; *p* = 0.005).

The height of the vertical jump of the control group rebounded slightly immediately after the game, 1 h after the intervention, and 24 h after the intervention but failed to return to its original level. Furthermore, there were significant differences between time points (*p* < 0.05), however, except for no significant difference between post-warm-up and 24 h post-intervention (*p* = 1.000). Although there was a significant drop in vertical jump height after CWI immediately, the recovery results showed an advantage at 1 and 24 h after the intervention, eventually returning fully to post-warm-up levels. There were no significant differences between 24 h after intervention and post-warm-up (*p* = 0.056), as well as between 1 h after intervention and post-warm-up (*p* = 1.000); there were significant differences between each time point (*p* < 0.05). The vertical jump height continued to rebound after the VFR and eventually returned almost to its original level.

### 3.2. Influence of Different Intervention Methods on Agility

As shown in Figure 3 and Table 2, there was a significant increase in response time after CWI immediately, which was significantly different from the other two groups (*p* = 0.004; *p* < 0.001). At 24 h after the intervention, the reaction time of the CWI recovered further and was significantly different from that of the CON (*p* < 0.001).

Significant differences were found between immediately after the game and the other four time points (*p* < 0.001; *p* = 0.005; *p* = 0.017). A significant difference was found between 1 h post-intervention and immediately after warm-up (*p* = 0.030; *p* = 0.024). The reaction times showed a slow recovery to the original level immediately, 1 h, and 24 h after CON, with significant differences observed between immediately after the game and immediately after warm-up, as well as between 1 h and 24 h after intervention. There were also significant differences between immediately post-intervention and after warm-up, 1 h, and 24 h after intervention (*p* < 0.001). The reaction times increased substantially after CWI immediately and returned rapidly to post-warm-up levels 1 h and 24 h after the intervention. There were significant differences among all five time points (*p* < 0.001; *p* = 0.001; *p* < 0.001; *p* < 0.001).

### 3.3. Influence of Different Intervention Methods on Dynamic Balance Ability

The dynamic balance score after CWI rebounded and showed a significant difference compared to CON (*p* = 0.021). At 24 h post-intervention, the dynamic balance score for the CWI rebounded considerably and was significantly different from the CON (*p* < 0.001).

As shown in Figure 3 and Table 2, except for no significant differences between 1 h post-intervention and 24 h post-intervention (*p* = 1.000), there were significant differences between each time point (*p* < 0.05). The dynamic balance score showed a slow recovery but did not reach the original level immediately, 1 h, and 24 h after CON. Significant differences were observed among the other time points (*p* < 0.05), except for no significant differences between post-warm-up and 1 h, as well as between 24 h after the intervention (*p* = 1.000; *p* = 0.116). The dynamic balance scores continued to rebound after CWI and eventually fully returned to post-warm-up levels. Furthermore, there was a significant difference between post-warm-up and immediate post-intervention (*p* = 0.017). Significant differences were also found between immediate post-game and post-warm-up, immediate post-intervention, 1 h, and 24 h post-intervention (*p* < 0.001). The dynamic balance score gradually recovered and returned to its original level after the VFR.

## 4. Discussion

This study aimed to investigate the effects of three recovery interventions, CON, CWI, and VFR, on lower extremity recovery following the simulated load of a basketball game in amateur basketball players. The main findings are as follows: (1) immediately after the game, all tested parameters significantly decreased with no differences observed between the groups; (2) immediately post-intervention, except for a significant improvement in dynamic balance score, CWI showed further decreases in other parameters; (3) at 1-h post-intervention, both CWI and VFR significantly improved jump height; (4) at 24 h post-intervention, CWI and VFR had recovered their parameters to pre-match levels, but CWI demonstrated better recovery effects. Additionally, the CON group showed lower levels in most parameters except for reaction time.

According to the relevant research statistics, an average basketball player spends approximately 34% of his time in fast-running jumps, and the average number of jumps at the end of each game exceeds 40 times. The results of this study showed that the vertical jump height of subjects decreased significantly after CWI compared to the other two groups, which is consistent with the findings of previous studies. Patterson et al. [40] explored changes in sports performance at CWI (10 °C, 20 min) immediately, 17 min, and 32 min later. The results showed that the vertical jump height of CWI decreased significantly by 17.5%. Similarly, Didehdar et al. [41] found that the instant vertical jump height of CWI dropped sharply by 37% when subjects underwent 5 °C for 15 min. Immediate hypothermic stimulation from cold therapy may lead to a decrease in enzymatic responses in the body, impeding cross-bridge interactions, and ultimately, resulting in reduced muscle performance [42].

However, we observed a 24% instantaneous decrease in vertical jump height after CWI, which differs somewhat from the results of the aforementioned study, and this may be related to the selection of subjects and the experimental design of this study, which focused on amateur basketball players who performed a CWI at 5 °C for 12 min. At 1 h and 24 h of intervention, the longitudinal height of both CWI and VFR recovered further and eventually returned to pre-competition levels. In addition, the CWI was more effective, with significant differences between the other two groups at 24 h after the intervention. Studies have shown that CWI can significantly reduce immediate muscle heat stress and heat loss [1,43].

Meanwhile, on the other hand, CWI can rapidly increase blood circulation over time, promoting the elimination of metabolites and inflammation, thereby accelerating the body’s recovery [44,45]. Although VFR is also effective in promoting blood circulation and speeding up recovery through vibratory rolling on the lower limb muscles [46], its long-term effects were not as significant as those of CWI in this study, which may be attributed to the specific VFR protocol used.

In this study, the subjects were only exposed to a 12-min session of 30 Hz VFR. While this VFR protocol may be more effective, as concluded in previous studies, there may still be room for improvement in the duration and intensity of the VFR intervention. A further analysis of different VFR protocols should be conducted in subsequent studies to identify the optimal protocol for different groups. A study by Didehdar et al. [41] found a significant positive relationship between muscle temperature and vertical jump height. A study by Bergh et al. [47] reached similar conclusions, with a 4.2% increase/decrease in vertical jump height for every 1 °C increase/decrease in muscle temperature.

Although the muscle surface temperature of the subjects was not measured in this study, we found through the subjects’ feedback that the subject’s somatosensory lower limb temperature was significantly higher than the VFR 1 h and even 24 h after CWI. A study [46] also found that the skin surface temperature began to increase rapidly 15 min after the cold therapy intervention, which may be the reason for the better effect after CWI at 24. Although the muscle surface temperature of the subjects was not measured in this study, we found through the subjects’ feedback that the subject’s somatosensory lower limb temperature was significantly higher than the VFR 1 h and even 24 h after CWI.

The results of the study found a substantial decrease in response time immediately after the CWI, with significant differences between both other groups, which is consistent with the results of some of the previous studies. Patterson et al. [40] showed a significant increase in subjects’ *t*-test (a measure of agility) time after 10 °C for 20 min after CWI. The exposure of the body to cold (cold steam or cold water, etc.) may have led to an increase in local stiffness and a decrease in agility as the temperature continued to decrease [48]. At the same time, some studies have produced inconsistent findings, with Evans et al. [49] measuring changes in agility qualities by three methods after subjects performed a dominant leg CWI at 1 °C for 20 min. There were no significant differences between CWI and CON. CWI did not significantly reduce the agility of the subjects compared to CON. After 24 h after the intervention, the agility of the three groups rebounded to the pre-competition level, and the effect of CWI was better, which was significantly different from that of CON. The gradual recovery of agility at 24 h after CWI and VFR was consistent with the results of the previous studies. The main reason may be that these two intervention methods improved the speed of blood circulation, accelerated the gradual elimination of metabolic substances and inflammation, and thus, promoted the recovery of agility.

In addition, the results of this study also found that the agility of 24 h after CON rebounded to the level of pre-game, which may be related to the agility test method of this study. Subjects were instructed by a computer to perform front-to-back, left-to-right response tests on a surface response area of only 1 m^2^ to assess agility. Due to the limitation of the test area, it may not be necessary for the subjects to carry out high-intensity lower extremity motion, so, it is easier for the agility of the subjects to recover to the pre-competition level after 24 h. Subsequent studies should be conducted to further confirm the validity and reliability of the test method in this study based on different agility test methods.

It was shown in this study that immediately after the intervention, the dynamic balance score of the CWI rebounded rapidly, with a significant difference between the CWI and the CON, this result differs from previous related studies. A study by Montgomery et al. [50] showed that performing 10 min of 12 °C CWI below the hip significantly reduced subjects’ dynamic balance, whereas performing partial CWI below the knee or ankle had no significant effect on dynamic balance. It can be seen from the above studies that although different ranges of CWI may have different impacts on the dynamic balance ability, relevant studies have not found the results of immediate dynamic balance ability recovery of CWI. The results of this study may be caused by the following three reasons: (1) The subjects recruited for this study have not been exposed to the Y-balance test before, so the learning effect may have a certain impact on the results of the dynamic balance ability test; (2) The participants’ joint relaxation and proprioceptive decline were caused by fatigue immediately after the game, thus leading, to the decline in joint stability. The cold-therapy intervention immediately after the game rapidly reduced the energy consumption of the body, improved joint stiffness, and activated the central regulatory mechanism, thus, promoting the recovery of the dynamic balance ability [51]. (3) Relevant studies have shown that cold therapy only affects the skin surface receptors but fails to affect the joint receptors that stabilize joints [52]. Therefore, after cold therapy, with the elimination of fatigue, the dynamic balance ability recovers. De Benito et al. [37] believed that VFR accelerated the recovery of neural pathways and improved joint proprioception, thus, promoting the improvement of dynamic balance ability.

Nevertheless, the study has several limitations that should be considered. Firstly, the absence of a specific cleaning period may have influenced the carryover effect, potentially impacting the observed outcomes. And, the simulated games may not fully replicate the real game situations. Additionally, the study’s small sample size and potential learning effects are additional limitations that should also be considered. Future research should incorporate a designated cleaning period, control for fatigue, include real-game scenarios, increase the sample size, and account for additional factors influencing recovery. Addressing these limitations would enhance the validity and applicability of findings in post-game recovery strategies for basketball players.

## 5. Conclusions

Throughout the paper, it was concluded that if amateur basketball players need to continue to exercise in training or after the game, it is recommended to use VFR for short-term recovery intervention. If training or competition is performed on alternate days, either VFR or CWI can be used for short-term recovery intervention, with CWI being the preferred choice. This study suggests that the short-term recovery of dynamic balance after VFR may be related to the above reasons. However, as the recovery process progressed, both CWI and VFR had a lesser impact on joint proprioception. The main reason for the recovery of dynamic balance ability may be attributed to the fact that both interventions, to some extent, accelerated blood circulation, facilitating the gradual elimination of metabolic substances and inflammation, and increased joint flexibility, thereby expediting the recovery of dynamic balance ability.

## Figures and Tables

**Table 1:** Participant demographic characteristics.

Variables		Subjects’ Profile
Age (years)		22.80 ± 0.84
Height (cm)		1.79 ± 0.04
Body weight (kg)		75.56 ± 6.59
Advantageous leg length (cm)		91.70 ± 4.37
BMI (kg/m^2^)		23.61 ± 2.34
Basketball years played (years)		4.40 ± 1.14
Number of training/competitions (times/week)		2.60 ± 0.89
Position of play	Point Guard	4
	Shooting Guard	2
	Small Forward	4
	Power Forward	0
	Center	0

**Table 2:** Changes in three types of physical fitness in different interventions at different times.

Vertical Jump Height	CON	CWI	VFR	Mean Range		
(cm)	Mean (SD)	Mean (SD)	Mean (SD)	Δ 1–2	Δ 1–3	Δ 2–3
post-warm-up	33.03 ± 2.12	33.15 ± 1.86	33.03 ± 2.04	0.119	0.003	0.122
immediate post-game	28.06 ± 2.29 a	27.90 ± 2.26 a	27.57 ± 2.14 a	0.159	0.485	0.325
immediate post-intervention	28.73 ± 2.18 ab	25.23 ± 2.48 #ab	29.18 ± 2.04 *ab	3.501	0.499	3.951
1 h after the intervention	29.46 ± 1.71 abc	30.15 ± 2.01 #abc	30.90 ± 1.96 &abc	0.687	1.437	0.751
24 h after intervention	30.90 ± 1.87 abcd	33.22 ± 2.13 #bcd	31.51 ± 2.14 *bc	2.327	0.608	1.719
RM ANOVA	Does it satisfy Mauchly’s test of sphericity? No (*p* = 0.032)			F (4.051, 56.710) = 32.202	Interaction (*p* < 0.001)	
**Reaction Time (s)**	**CON**	**CWI**	**VFR**	**Mean Range**		
	**Mean (SD)**	**Mean (SD)**	**Mean (SD)**	**Δ 1–2**	**Δ 1–3**	**Δ 2–3**
post-warm-up	24.20 ± 1.21	24.33 ± 1.23	24.13 ± 1.60	0.133	0.067	0.200
immediate post-game	28.20 ± 2.73 a	27.60 ± 2.38 a	27.80 ± 1.66 a	0.600	0.400	0.200
immediate post-intervention	26.53 ± 1.73 a	29.47 ± 2.47 #a	25.27 ± 2.12 *b	2.933	1.267	4.200
1 h after the intervention	25.20 ± 1.42 bc	25.40 ± 1.84 bc	24.87 ± 1.55 b	0.200	0.333	0.533
24 h after intervention	25.73 ± 1.83 b	23.40 ± 1.80 #bc	24.13 ± 1.96 b	2.333	1.600	0.733
RM ANOVA	Does it satisfy Mauchly’s test of sphericity? No (*p* = 0.668)			F (8, 112) = 8.095	Interaction (*p* < 0.001)	
**Dynamic Balance**	**CON**	**CWI**	**VFR**	**Mean Range**		
**(** **%** **)**	**Mean (SD)**	**Mean (SD)**	**Mean (SD)**	**Δ 1–2**	**Δ 1–3**	**Δ 2–3**
post-warm-up	83.02 ± 7.26	82.96 ± 7.67	82.40 ± 7.22	0.060	0.622	0.562
immediate post-game	75.25 ± 8.06 a	76.37 ± 7.83 a	76.09 ± 7.64 a	1.122	0.838	0.284
immediate post-intervention	78.34 ± 7.90 ab	80.31 ± 8.65 #ab	80.18 ± 7.47 ab	1.969	1.842	0.127
1 h after the intervention	80.26 ± 7.40 abc	82.40 ± 7.95 bc	81.67 ± 6.62 b	2.146	1.414	0.732
24 h after intervention	80.59 ± 7.32 abc	84.68 ± 7.73 #bcd	81.88 ± 7.34 b	4.097	1.301	2.796
RM ANOVA	Does it satisfy Mauchly’s test of sphericity? No (*p* = 0.015)			F (4.183, 58.555) = 3.741	Interaction (*p* = 0.008)	

Note: CON = control group; CWI = cold water immersion group; VFR = vibration foam rolling group; a. significant difference within-group for post-warm-up in each group compared to other moments (immediate post-game, immediate post-intervention, 1 h post-intervention, 24 h post-intervention); b. significant difference within-group for immediate post-game in each group compared to other moments (immediate post-intervention, 1 h post-intervention, 24 h post-intervention); c. significant difference within-group for immediate post-intervention in each group compared to other moments (1 h post-intervention, 24 h post-intervention); d. significant difference within-group for 1 h post-intervention in each group compared to 24 h post-intervention; #—significant difference between CON and CWI; &—significant difference between CON and VFR; *—significant difference between VFR and CWI.

## Data Availability

Data are available on request due to the restriction of ethics.
